# Supplementary material for: MetaRibo-Seq measures translation in microbiomes
Source: Nat Commun. 2020 Jun 29;11:3268. doi: 10.1038/s41467-020-17081-z (PMC7324362; doi:10.1038/s41467-020-17081-z)
Supplement: Supplementary file 10 — Supplementary Data 7 [file 41467_2020_17081_MOESM10_ESM.zip › File2/Confidence_High_Taxonomy/347881_out.krona.html]

Javascript must be enabled to view this page.

members
magnitude
magnitudeUnassigned
count
unassigned
taxon
rank

347881\_out

3

3
2
superkingdom

phylum
976
3

class
200643
3

3
order
171549

family
171552
3

genus
838
3

1262923
species

SRS015663\_contig\_number\_5828SRS043768\_contig\_number\_5901SRS051031\_contig\_number\_28046
3
